# Prisoners on prisons: Experiences of peer-delivered suicide prevention work

**DOI:** 10.1177/26326663231172023

**Published:** 2023-05-25

**Authors:** Gillian Buck, Philippa Tomczak, Paula Harriott, Rebecca Page, Kate Bradley, Mark Nash, Lucy Wainwright

**Affiliations:** 1 Department of Social Work and Interprofessional Education, University of Chester, Warrington, UK; 2 University of Nottingham, Nottingham, UK; 3 240358 Prison Reform Trust, London, UK

**Keywords:** suicide, peer support, prison, mental health, wellbeing

## Abstract

Prison suicide is a global concern, with rates consistently exceeding those in non-incarcerated populations. Prisoners deliver (suicide prevention) initiatives in jurisdictions around the world. As part of a research project seeking to foreground prisoner voices in criminological knowledge, former prisoners and academics coproduced an innovative, retrospective examination of peer-delivered prison suicide prevention in England. Our collaborative, autoethnographic research design involved focus group discussions and co-authored outputs. We offer fresh perspectives on peer-delivered suicide prevention, revealing overlooked limitations including traumatisation through ‘volunteering’. Findings include: the riskiness of prison peer support; inconsistencies in training and conditions; the importance of (supported) peer provision; and proposals for safer service development.

## Introduction

Prisoner participation in prison life has become more prominent in European political agendas (Brosens, 2019). Peer support work – undertaken by prisoners with prisoners – increasingly forms part of prison suicide prevention strategies in countries including the United Kingdom (Buck, 2020), the Republic of Ireland (Griffiths and Bailey, 2015), France (Auzoult and Abdellaoui, 2013), Canada (Hall and Gabor, 2004) and Australia (Hinde and White, 2019). Peer approaches can offer social support (Adair, 2005) and opportunities to acquire new skills and give back (Edgar et al., 2011). Consequently, peer supporters can begin to resist negative labelling and forge desired selves by ‘doing good’ (Perrin et al., 2018: 759), potentially supporting desistance and mental well-being (Nixon, 2020a; Einat, 2017). Yet, significant concerns surround the exploitation of imprisoned ‘volunteers’ providing emotional labour for free (Hinde and White, 2019). Peer support has been driven ambivalently: by criminalised people seeking responsive methods and by neoliberal concern with cost savings (Buck, 2020).

Research into peer-delivered (suicide prevention) initiatives principally draws on accounts from serving prisoners (Perrin and Blagden, 2014). Longitudinal research is required (Griffiths and Bailey, 2015) and no studies have retrospectively examined prison volunteering *with* former volunteers. Prisoner authorship can expose conditions, prompt debate on reform, raise consciousness and assist people to process their confinement (Nellis, 2002). Prisoner accounts may support structural change, for example, Wildeblood (1955) added momentum to campaigns to decriminalise homosexuality. Despite efforts to position prisoners as agents in prison scholarship (Crewe and Bennett, 2012), there is limited peer-reviewed literature (co-)authored by people with lived experience of punishment (Bosworth et al., 2005; Weaver and Weaver, 2013).

Addressing gaps both in prison peer support knowledge and prisoner-authored scholarship, this article examines the experiences of five former prisoners who volunteered as emotional peer supporters whilst imprisoned in England. Our innovative co-authorship approach reveals overlooked challenges of prison peer support, centring former prisoners’ suggestions for improvement. First, we explore the literature on prison suicide, self-harm and peer support. Next, we outline our participatory approach to gathering and analysing qualitative data. Our findings provide a case study illuminating peer support limitations in prison and post-release. We highlight (i) high levels of need volunteers can face, (ii) inadequate resources to meet prisoner needs and (iii) opportunities for policy makers and providers to work with peer supporters to improve policy and practice.

## Prison suicide and peer support

Suicide is the act of intentionally taking one's own life (Mind, 2020), although confused and mixed intentions are seen in self-inflicted prisoner deaths (Walker and Towl, 2016). Prisoners who self-harm and/or have histories of suicidal ideation are at increased risk of suicide while incarcerated (Favril et al., 2020). Across jurisdictions, prisoner suicide rates exceed those amongst comparable groups in the general population (Zhong et al., 2021). In western Europe, suicide rates exceed 100 per 100 000 prisoners (Fazel et al., 2017), generating very significant harms and costs (Tomczak, 2018).

High rates of suicide and self-harm in prison are a concern internationally. In October 2021, the UN Special Rapporteur on extrajudicial, summary or arbitrary executions identified deaths in custody amongst his four priority areas, stating: ‘most deaths in custody are preventable’^1^. Prison suicide reduction has been designated a priority activity by the World Health Organisation (2007) and England and Wales’. Nevertheless, suicide prevention practices in prisons remain ‘tenuous’, requiring empirically underpinned models of training and intervention (Cramer et al., 2017).

Prison suicide prevention analyses cannot be separated from broader questions including how many people we imprison, who is sent to prison and how prisons are resourced and run (Aitken, 2022). In many countries, imprisonment rates have increased dramatically over recent decades (Wacquant, 2010). In our case study jurisdiction: England and Wales, amidst static or falling crime rates, the prison population has risen by 70% in 30 years, maintaining the (second) highest imprisonment rate in Western Europe (Prison Reform Trust, 2018). Justice Secretary Grayling's benchmarking ‘efficiencies’ from 2012 generated the largest staff reductions in prison service history, leaving fewer, less experienced staff managing more prisoners (Prison Reform Trust, 2018). The voluntary early redundancy scheme also reduced prison staff levels, constraining the capacity to foster relationships and escort prisoners to health appointments (Howard League, 2017). Staffing ‘efficiencies’ correlate with increased deaths. Between 2012 and 2016, prison suicide rates more than doubled. 2016 saw a record high of 122 prison suicides (MoJ, 2017), followed by consecutive record rates of self-harm in 2017, 2018 and 2019 (Tomczak and Bennett, 2020).

In March 2018, U.K. government proposals to address prisoners’ mental health increased grant funding to the Samaritans charity, for their ‘Listeners’ peer support scheme (Tomczak and Bennett, 2020). Samaritans train prisoners nationally to provide confidential emotional support to other prisoners, aiming to reduce distress which might lead to (thoughts of) self-harm and suicide (Samaritans, 2020b). Local Samaritans volunteers train serving prisoners in listening skills, confidentiality and the law concerning suicide, following which prisoner Listeners provide 24-h confidential support to fellow prisoners and are escorted to callouts by officers (Foster and Magee, 2011: 13). The Listener scheme is the foremost prison peer support scheme, with Listeners in 112 prisons across the United Kingdom and Ireland (Samaritans, 2020a).

There are also many other peer support providers. In England and Wales, the Prisons Inspectorate (2016) listed 29 examples of peer support including Listeners, mentors and advisors. Some prisons also organise prisoner volunteers themselves to support emotional well-being (Telfer et al., 2021). Overseas, the Irish Red Cross organises volunteer prisoner peer educators to enhance health (O'Sullivan et al., 2020) and initiatives in Canada and France have introduced peer-to-peer programmes to prevent prisoner suicide (Brosens, 2019). Prisoner volunteers can uniquely complement statutory services, given their ability to reach prisoners who may see talking to staff as violating the ‘inmate code’ (Perrin, 2022). Benefits of the Listener scheme include support for prisoners in distress and reduced pressure on prison and health professionals (Foster and Magee, 2011). Listeners have also reported job satisfaction, empowerment and personal growth (Dhaliwal and Harrower, 2009). The benefits of peer support schemes more generally include support for prisoner mental health and deepened pro-social bonds (O'Sullivan et al., 2020; Telfer et al., 2021), yet peer supporters in prison also face significant challenges.

Prisoners who adopt peer support roles can be viewed with suspicion by inmates and the work may be burdensome, resulting in diminished confidence or burnout (Jaffe, 2012; Perrin, 2022). Peer supporters work amidst very high levels of need and limited statutory service provision. In England and Wales, 70% of male prisoners have an underlying mental health need but only 10% receive mental health services (House of Commons Committee of Public Accounts, 2017). 46% of women prisoners have attempted suicide at some point, yet only 28% said it was easy to see a mental health worker (Prison Reform Trust, 2021). More than half of prisoners were negative about the quality of prison healthcare (Prison Reform Trust, 2021). Imprisonment itself can induce psychological disturbance (Liebling, 2007), particularly as jails are ‘plagued by drugs, violence, [and] appalling living conditions’ (HM Chief Inspector of Prisons, 2016: 7). Despite this context, the U.K. government ‘does not know how many people in prison have a mental illness, how much it is spending on mental health in prisons or whether it is achieving its objectives … [improving mental health in prison will therefore] require a step-change in effort and resources’ (National Audit Office, 2017: para 1–2).

Amidst these conditions, peer provision holds the potential for exploitation, particularly during austerity. Volunteers may be required to complete duties previously undertaken by paid employees (Nixon, 2020b; Verhoeven and Van Bochove, 2018). In HMP Elmley, for example, prisoner violence reduction volunteers filled ‘the gaps left by the inconsistent staff presence’ (HMIP, 2016: 13). In coercive detention, ‘volunteer’ power relations are also problematic. Every element of a prisoner's behaviour is assessed to inform decisions about entitlements. The Incentives and Earned Privileges (IEP) scheme in England and Wales determines significant matters such as whether prisoners can wear their own clothes, have a TV, have visits, or be released (as discretionary parole decisions apply to some sentences). Prisoners are acutely aware of this scrutiny, which influences choices, including whether to volunteer and/or speak openly about any problems with ‘volunteering’. Moreover, these discretionary processes are affected by structural discrimination, with, for example, IEP decisions about privileges perceived by ethnic minority individuals as unfairly applied (ZMT, 2021). Qualitative, (auto)ethnographic work has revealed the value of insider accounts of prison suicide and related health cultures (Nixon, 2022), yet the focus of studies has often been prison officers rather than imprisoned volunteers (Ricciardelli et al., 2020).

## Research design and methods

Our study employed a rigorous qualitative design, guided by an interpretivist philosophy (Bachman and Schutt, 2014), to consider how peer support was meaningful to participants. Most research about marginalised people is done *by* those who are not marginalised (Brown and Strega, 2015), which can result in participants’ experiences of pain ‘being used, manipulated and repackaged’ often without their knowledge or full consent, leaving minimal impacts on the lives of criminalised people or structural conditions (Booth and Harriott, 2021: 199). To avoid these omissions, we included formerly imprisoned peer supporters as co-investigators, extending the rich history of prisoner inclusion in criminology. For example, the tradition of ‘convict criminology’ elevates lived experience perspectives in knowledge production to sharpen analytical foci (Earle, 2018). We purposely included male and female co-researchers to overcome the historic marginalisation of women in (co)producing academic knowledge (Earle, 2018).

Our research question initially came from a former prisoner co-author, who felt that little criminological literature represented the realities of prison life. She theorised that writing *by* people with prison experience could lead to new knowledge and suggested a study bringing former prisoners and academics together to explore prison peer support work. The academics suggested ‘participatory action research’ (PAR) methodology. Participatory approaches belong to a ‘transformative paradigm’ (Mertens, 2009) in which the agency of participants is emphasised, and researchers and communities work together for social transformation. The researcher's role is to share information in ways that empower, aiming to redress inequalities (Mertens, 2009: 319). PAR assumes that people impacted by a topic should be co-researchers (Valenzuela, 2016), blurring researcher/ participant distinctions to create a democratic inquiry (Marshall and Rossman, 2011: 23) so that practice can be improved (McCutcheon and Jung, 1990: 148). Such collaboration can be time-consuming, but also educational and worthwhile.

**Figure 1.**
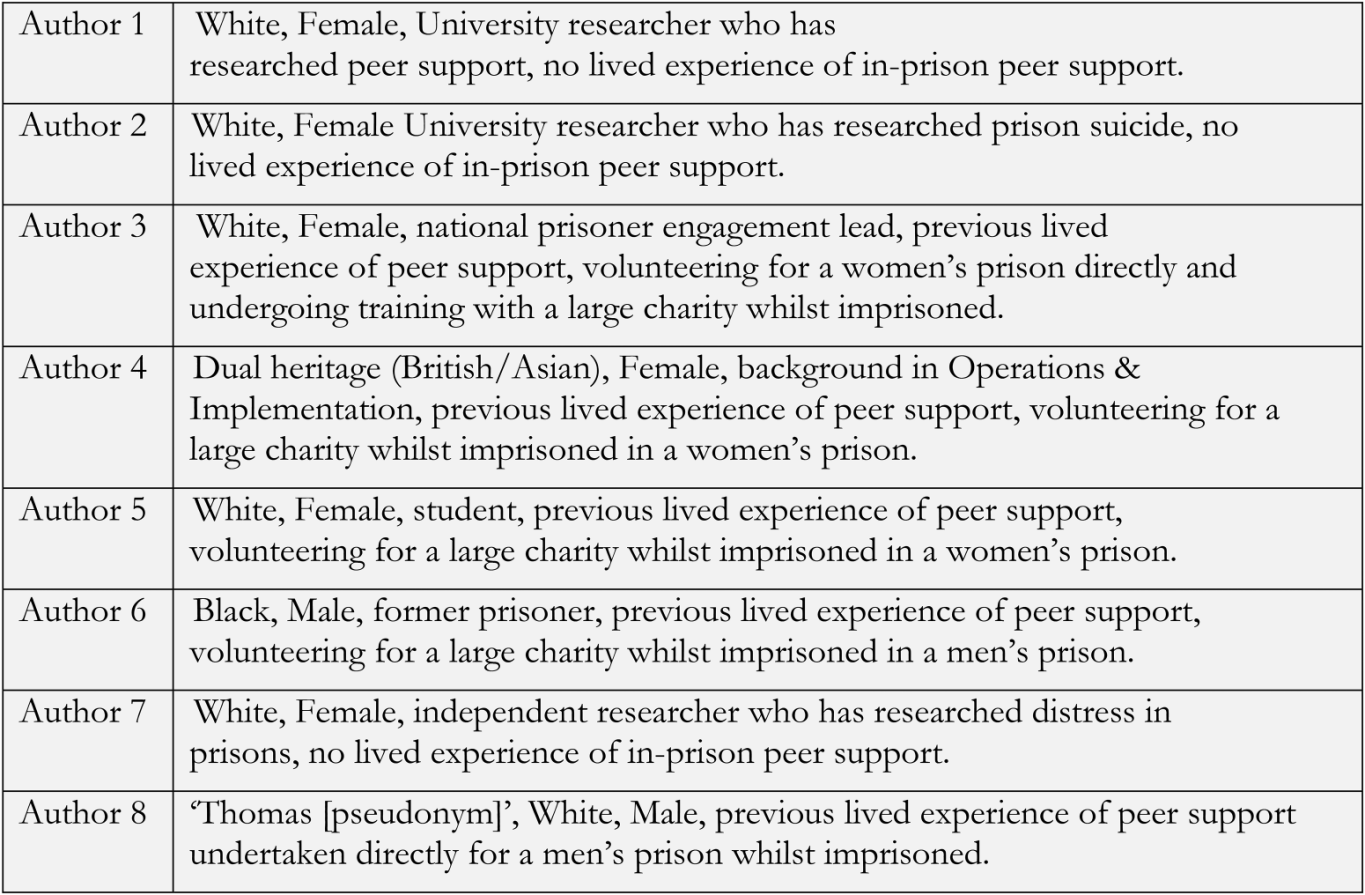
Authorship team.

Co-authors were identified through the *Prisoner Policy Network* (PPN), hosted by the *Prison Reform Trust*. The PPN is a network of (ex-)prisoners and allies working to include prisoners’ experiences in national policy development. Following University ethical approval for our study, a PPN representative invited a purposive sample of people connected to the network to participate. The inclusion criterion was experience in prison suicide prevention work. People released on licence were excluded due to the Prison Service research moratorium amidst the pandemic (MoJ and HMPPS, 2020). Co-researchers therefore had the benefit of some years since imprisonment to reflect, but limited experience during the pandemic – when well-being in prisons has worsened (Hewson et al., 2020). Our team assembles eight authors: three with academic knowledge of ‘criminal justice’ and five with lived experience of imprisonment. We occupy diverse, fluid identities (see Figure 1). Whilst we reflected *on* diverse identity positions, our sample was too small to reflect on how intersecting differences (e.g., gender, ethnicity, age) impacted peer support or establish significant differences across groups (e.g., male/ female). These would be useful foci for future research.

We adopted Ledwith's (2016) ‘cycle model’, which provides a template for critically considering social issues in partnership and taking action. Steps included: (i) ‘Being’, that is, noticing the issue of prison suicide, (ii) ‘Problematising’; group discussions of what is happening in prisons and why, (iii) ‘Conscientisation’; considering the implications of this issue and what should be done about it – via focus groups and writings, (iv) ‘Action’; co-writing this article, (v) ‘Making sense’; discussing desired outcomes from publications to planning next steps together, (vi) Communication; translating our work to stakeholders including voluntary organisations, prisons, policy makers and prisoners.

Our methods included (i) focus groups, which can generate in-depth discussions and place multiple perspectives in dialogue, potentially providing collective power to marginalised people (Liamputtong, 2011) and (ii) collaborative autoethnography. Autoethnography is a written method in which people describe the personal experience (auto) to understand cultural experiences (ethno) and systematically analyse them (graphy) (Gant et al., 2019). Collaborative autoethnography involves sharing accounts of personal experiences to support deeper analysis. This can result in highly personalised accounts from (frequently unheard) groups and offer ‘novice’ researchers an opportunity to deepen learning and develop with others (Gant, 2022; Gant et al., 2019).

Our initial focus group in September 2020 was attended by all authors, via online videoconferencing. One co-author prompted the discussion, asking how former prisoner authors learned they could volunteer in suicide prevention and what motivated them. The ensuing 100-min discussion was ‘unstructured’, to centre those most impacted. With the informed consent of the group, the discussion was audio-recorded and transcribed verbatim. After this group, all authors were invited to write reflections so that one month later, we could discuss these together. The writing process was explained at the end of the initial group:

*Academic researcher:* In a previous project like this, I wrote a draft and sent it to other authors. One co-author wrote her own sections, another spoke to me over the phone and said, ‘this needs to go in’, or ‘I don't agree with this’. So, tell me how you are comfortable working. You might want to call or just write your ideas …. You might want to write a poem or do something visual; we’re open to suggestions …. We have another three (optional) group dates planned if you want to test ideas. We’re learning here too, so if you’ve got ideas, tell us. You’re our co-authors.

Most group members were more comfortable talking than writing, so the academic partners wrote up some spoken reflections and linked these to others’ written reflections and wider phenomena. For example, recollections of peers responding to extreme distress with little support were linked to notions of secondary and vicarious trauma. The group then discussed the need for prisons and charities to recognise and minimise this potential. Reflection methods were deliberately adaptable to facilitate the inclusion of diverse competencies (Watson and Fox, 2018). One co-author reflected:

Feeling comfortable and having a safe space is paramount to extrapolating peoples’ lived experiences and not enough researchers allow time for this. Lived experience participants are often just “used” as we know all too well. *This is one of the main reasons I usually decline to take part in research.* (Emphasis in original).

Given that recalling experiences of imprisonment can be traumatic, we urge other researchers to consider the adaptability of research design to individual needs.

Additionally, the academic researchers analysed data thematically (Braun and Clarke, 2021), which involved coding and theme development. Coding involved reading the focus group transcript and written submissions, highlighting material and adding comments; interpretation involved creating clusters from codes and interpreting meaning in relation to the research questions. For example, codes such as ‘risky environments; high expectations; lack of care’ were grouped into themes, for example, ‘riskiness of peer support’, which was interpreted in terms of psychological well-being. Written interpretations were emailed to co-authors, who wrote remarks or made verbal suggestions. Four hour-long online meetings took place between October 2020 and January 2021, to clarify themes as a group and acknowledge any distress. In this way, the data collection and analysis were collaborative and recognised needs (Chevalier and Buckles, 2013). From April 2021 to August 2022, the paper underwent peer review by two social science journals and both rejected it after requesting revisions. Authors collaboratively planned amendments via email and videoconferencing. The first journal wanted more engagement with critical suicidology theory (e.g., White, 2017). However, we did not fully implement suggestions as they reduced the prisoner-led focus and the revised work was rejected. A reviewer for the second journal made negative assertions about co-authors’ level of training and ability to contribute to knowledge production. After strengthening our defence of subjectivism and autoethnography, the article was rejected again. As such, we have learned first-hand that peer review forms part of the knowledge hierarchy (Jaffe, 2017), which can be dismissive of alternative knowledge from groups that are rarely visible as authors. Despite resistance, we remained committed to defending our innovative approach and sharing our valuable data.

The power imbalance between researchers and (former) prisoners can result in exploitation (Dupont, 2008). We minimised this risk by inviting open discussions about our different positions, ensuring informed choice about accredited authorship/anonymity, sharing knowledge about research philosophies, methods and publishing norms, and valuing the ‘deep wisdom’ that emerges from reflection on experiences (Booth and Harriott, 2021). We also ensured that the team comprised more former prisoners than professional researchers and ensured that formerly imprisoned co-researchers were paid. Despite concerns that payment can induce participation and limit abilities to make uncoerced decisions, research participation decisions are based on multiple factors and incentives to communicate the worth and status of all persons (Hanson et al., 2012).

Decisions about anonymity were informed by the ‘hands-off our stories’ principles (Costa et al., 2012), which invite consideration of the risks of self-disclosure, given that experiences may be appropriated for organisational interests:

- Participation is voluntary. You can always say no.
- Ask yourself, who profits from you telling your story?
- What purpose does personal story-sharing serve?
- How do large organisations use stories to make material change?
- Storytelling as an exercise of labour/ work. Do you get paid?
- The internet lasts forever. Because of the technology available today, your interview or story will likely be accessible to the public for a very long time. That includes future employers and landlords (Costa et al., 2012: 93).

These principles were integrated into information materials and discussed in focus groups. Seven authors made an informed decision to attach their names to this article and one person remains anonymous. Quotations in the analysis are deliberately not attributed, offering some confidentiality whilst recognising authors for their contribution.

## Findings

Although peer support can complement statutory services (Perrin et al., 2018), former prisoner reflections were dominated by the mental health crisis in prisons and the high levels of distress and self-harm that volunteers faced *as a norm*, with little structural or personal support. Sub-themes included (i) the riskiness of peer support; (ii) inconsistencies in training and working conditions; and (iii) the value of peer support*.* We explore these sub-themes, then propose co-created recommendations for safer service provision.

### The riskiness of prison peer support

Evaluations of prison peer support (e.g., Perrin and Blagden, 2014; Samaritans, 2011) rarely acknowledge differences between volunteering whilst imprisoned and community volunteering. There are distinctive risks of prison volunteering including intimidating environments, high expectations and fraught relationships. For example, one former prisoner reflected on their induction:

I don’t know this space. I don’t trust you as a prisoner. I don’t trust you as a member of staff … my experience of people in uniform has not been about trustworthiness, it's about bullying and authority and lack of respect.

Prisoners often have histories of inconsistent or abusive relationships with authorities (McNeill, 2013). When working as peer supporters, authority figures become simultaneously jailers and colleagues, often leading to ambiguity and mistrust. This entails ‘dirty emotional labour’ (Nixon, 2020b): managing cynicism and mistrust from prison officers and other prisoners *in addition to* the work itself. Another former volunteer identified additional pressures:

I didn’t push to get involved because often [volunteers] were asked to pass drugs around the wing. There was all the manipulation and bullying that went on behind the scenes.

Whilst studies note that prisoner peer supporters can be bullied or pressured to pass drugs, phones or information (e.g., Perrin, 2022), it remains unclear if or how prisons and charities are managing these risks, leaving peer workers structurally vulnerable. In addition to the challenging environment, volunteering took a physical and emotional toll:

[Officers] would come and get you out of your cell [to listen] at any time, three, four AM, but they expected you still to go to work at half-past seven to eight AM. I was getting called up regular and … even though I was doing something for the prison as a listener until six AM, I had to go to work or they wouldn’t pay me …. Between the officer picking you up at two AM and carrying you to the Listener suite^2^ – all of a minute – that's … what they call their support: “Are you okay?” That's it! And then bang you back up … there was no aftercare … everyone was being used – it's what you can do for the system, not what the system can do for you ….

*Former-volunteer response: If you got a minute, you were loved more than I was! I was asked if the caller was not in a good place, as that might cause [the prison] a problem, but no one asked me.*

Ideally, Listeners are offered weekly or monthly offload sessions by the Samaritans charity, but the regularity of these meetings varies (Foster and Magee, 2011) and our study noted that prison officers were too frequently present in such meetings when they did take place. Volunteers can be left to undertake acute suicide prevention work in the middle of the night, in the absence of support. They could also be required to go to work the next day with very limited rest. As a result, volunteers often felt used and unsupported, as illustrated in this extended reflection from a former volunteer:

Someone wants to see me in her room, which is pitch black at one PM … she rises, and crying, hugs me tightly, this rarely happens in prison. Suddenly, her body turns to a dead weight, and we fall to the ground. I am covered in blood; she has cut both her wrists. I shout the officers as I can’t reach the bell; no-one comes for a couple of minutes. Prisoners come rushing first, it is five minutes before any officers come. I am trying desperately to wrap her wrists in bedsheets. When the officers arrive, they grab me and fling me against the wall while they attend to her. She is screaming, they drag her out and off the wing, not an ounce of care. I remember a trail of blood on the landing. There are no officers on the wing now. I am left covered in blood, hoping she is ok. An officer returns and I am locked in my dorm; the officer asks if I am ok as we walk *– “I don’t know! Is she ok? Where is she? Is she still alive?*” … the response is “*I don’t know”*. I am locked in for three hours with the rest of the wing for security purposes. That was it, no: “do you need to speak to anyone? do you want a quick shower?” Not even the smallest aftercare. I’m left thinking *maybe it was my fault; did I say the right thing?* These questions will remain unanswered in my head …. This event will always stay with me, but it needs to be shared – for learning and development. I advocate peer support in prison 100%, but to be done safely, ‘support for support’ is needed; like support groups or one-to-one reflection – activities that cost little but have substantial positive impacts. If organisations want to utilise prisoners, they need to acknowledge their responsibility to support …. This is people's lives and mental health we are talking about; it *has to* be taken more seriously.

This narrative indicates a lack of regard for the human impact of this work. Despite her first-responder actions, which left her blood-soaked, shocked and distressed, the Listener was treated as a risk by prison staff and her emotional and physical needs were overlooked. This omission might also take place outside formal volunteering, as some prisoners were called to undertake suicide prevention roles without any training or organisational support:

I think I was unofficially blocked from becoming a Listener but [the prison] used me alongside the Listener. So, people were put in with me who had attempted suicide. No consent, no training, just my [cell] mate dragged out in the middle of the night, someone else put in. It was only the third, fourth, fifth time it happened I realised that I was being used unofficially.

Other group members asked questions of this speaker:

*Former prisoner co-author: How did you get identified as the person they thought could cope with that?*

I’ve no idea, my Personal Officer maybe? I was always quite reasonable, and I wasn’t bullying people or taking tobacco. I don’t know, it was never broached with me.

*Former prisoner co-author: Never a conversation – ‘why don’t you do this’?*

No

*Academic co-author: Did they say ‘do you want to make it official? Do you want to do the training’?*

They already had an official Listener. I don’t know if they just didn’t trust him … or couldn’t keep taking cellmates out of his cell in the night, so they just did it to me.

Former prisoner co-author: And when they threw somebody in who attempted suicide, what were you expected to do?

It was just “pad mate, get your kit together, you’re moving”, someone would come in and I would just chat with them, I’d talk to them all night and just talk it through ….

*Former prisoner co-author: They do that a lot, [officers]; pick out the nice people and put people with you … they used to try and put girls who were guilty of offences against children in my room because they knew I’d be a reasonable person. They’d say: “take them to dinner”, without telling me why, and possibly putting me at risk!*

This interaction revealed an ‘overspill’ of peer support work, wherein prison officers informally recruited ‘reasonable’ people to care for distressed, unwell and vulnerable prisoners without disclosure or follow-up. This may have been a consequence of peer support work changing officers’ expectations and uncovers a further significant gap in support, especially given that prisoner volunteers live in their working environment:

Former volunteer: I remember going out to the girls who say they’re going to kill themselves and waiting for breakfast the next morning … searching for these girls to check they’re alive and the anxiety is stressing me.

*Academic co-author: That makes me feel so much empathy because working in psychology, I felt that too. I used to go home and worry about that person that I’d seen at the end of that shift …* *[but] what we had was a supervisor, a trained team, a home to go to and safe surroundings. It just dawned on me that* I found it hard, and I had all of that. *What must that be like for somebody who isn’t with their family, who doesn’t have a supervisor, who has got their own trauma … every single day? (Emphasis in original)*

Suicidality is recognised as one of the most anxiety-provoking scenarios for helping practitioners (Rudd et al., 2008). Those responding to self-harm often experience anxieties that can impact their psychological well-being and negatively affect service delivery (Walker and Towl, 2016). In professional settings such as psychology, it is recommended that workers are supervised by someone with clinical and supervision expertise (Rudd et al., 2008), yet this safeguard appears to be rare or absent for prisoner peer supporters.

“***They just used to put us in the room with them”: Inconsistencies in training, working conditions and support.***

Peer supporters often navigated high expectations and minimal structural supports. The following reflection indicates the extreme distress prison volunteers could be faced with, being inappropriately substituted for professional healthcare rather than complementing statutory provision:

I got called down to the Seg^3^ and a woman was in a very, very, VERY bad way. She was just absolutely psychotic, I don’t even know the right words for it, I don’t want to say anything offensive. But she just kept taking her clothes off, screaming, shouting, making accusations about me, saying that she’d only speak to me and not my friend because I was Black and my friend was White, when I’m very clearly White, and we just stood there …. And I just always remember thinking, *what THE HELL do I do?* This was a really severe thing.

This task was inappropriate for peer supporters, but co-authors believed that they were expected to give prison officers ‘a few hours break’, amidst severely constrained mental health services and secure beds (Tomczak, 2022a). Working with acutely distressed people is challenging for trained, experienced, and supported healthcare professionals, but peer supporters enter these situations whilst dealing with their own incarceration and with very little preparation. One former prisoner reflected:

The [volunteer] training, how simple it was, was a disgrace really when you think about the turmoil that's going on in remand prisons, I mean, *absolute turmoil*. I’ve never seen self-harm in my life to that extent, I saw girls with plasters down their faces where they cut themselves open … it was a really, really, highly volatile environment. It didn’t feel like anybody was in control … just like ‘act tough’, like you know what you’re doing. And then I went to this training and the simplistic way they were discussing it all, I’m thinking ‘*You lot are crazy* if you think I’m doing this’. So, I did the training. And then I said I didn’t want to volunteer ….

Another co-author who did go on to volunteer added:

I concur with what others are saying … I internalised a lot. I very much compartmentalised it … The people that are really on the edge, *just cut themselves*, you go in and you’re not equipped … And I think it's unfair. We’re not being equipped by the people asking us to help. And you’re not *not* gonna do it because those people need that, but … it was really difficult for me, I felt like I was taking it all on board … It's too heavy. I couldn’t carry everyone's stuff. It was too difficult …. People study for years to ensure they are ready to do work such as this, with mandatory concentration on self-care, something that seems missing in peer support.

There is clear potential here for secondary or ‘vicarious’ trauma. Secondary trauma describes how those listening to experiences of trauma can experience post-traumatic stress disorder symptoms, vicarious trauma refers to how engagement with traumatic material may cause a disruption in the helper's view of self, others, and the world (Bober and Regehr, 2006). Anyone engaging empathically with traumatised individuals can be at risk of distress and impairment due to indirect exposure to traumatic material (Evces, 2015) and recent research has identified that despite benefits, prison peer mentoring can create trauma for mentors by having them re-live distressing experiences through discussions (Henderson and Meek, 2022). Whilst researchers have noted secondary and vicarious trauma impacting diverse criminal justice staff (Huggard et al., 2017; Newman et al., 2019; Perez et al., 2010), too little attention has been paid to prisoner volunteers. Without training and development structures that recognise and mitigate this phenomenon, there is a risk of harm to peer supporters and those they seek to support. The gap in support for prisoners was recognised by the group:

If you went to work and one of your teammates said, ‘I need to talk to you because I want to kill myself’, there would be uproar in the office … you know, ‘do you need counselling for that traumatic incident?’ … [I witnessed something traumatic in a community workplace, and] I was allowed to have time off work, they offered trauma counselling, and a short break. And in my head, I’m thinking, this is minimum trauma to the things that I’ve seen in jail and in my life … I just think there's this totally different reaction to people witnessing trauma when in the community than when we’re witnessing trauma as prisoners (Former prisoner co-author).

Co-authors were uncertain how much external organisations knew about the extent of trauma they faced. One former volunteer reflected:

I understand that Samaritans can be contacted for support having read their website, however, *in prison* we did not know this, or how to do this, or when, or even if we are allowed …. You have HMPPS (the Prison Service), Samaritans and the individual volunteer, all working in different ways.

Because volunteers are often trained (and ostensibly supported) by charities outside prison, yet transported and restricted by prison officers, the lines of management and accountability are disconnected. This is particularly concerning given that peer support is a major plank of prison suicide prevention.

Former volunteer: Once they didn’t get the Listener in time, and he cut his wrists. There was no observation. By the time the Listener got there they had to press the bell and say he's dead … There is no due care and attention or follow-up that could have prevented that from happening … it's just become normal. Seeing someone with mental health problems in prison is normal now.

When someone dies by suicide, it can have significant impacts on (volunteer) workers including intense reactions, emotional distress, sadness, blame, low-morale and anger (Powers, 2017; Whisenhunt et al., 2017). In their study of young men in prison, Hales et al., (2015) found significantly higher levels of psychiatric morbidity and suicide-related behaviours amongst those who had witnessed a peer's suicide-related behaviour. However, with support, posttraumatic growth may be promoted (Whisenhunt et al., 2017). Bradley (2017) advocates a trauma-informed approach, wherein all staff working with incarcerated populations are trained to consider the role trauma plays and individuals are supported to avoid re-traumatisation.

### The importance of peer support and listening

Whilst there is evidence of positive effects of peer support on prisoner well-being (Nixon, 2020; Perrin et al., 2018), our research design included former (not serving) prisoners, facilitating reflection beyond the all-encompassing and punitive environment of prison. This enabled people to critique more freely than whilst imprisoned. It also facilitated hindsight and, in some cases, changed perspectives:

I’m quite emotional now, but at the time you just compartmentalise it, [suicide behaviour] is normal, so they rely on the strength of characters of the prisoners … it would play on my mind when I was on my own, but day-to-day it becomes the norm …. Looking back, I probably realise now that I’ve been taken advantage of, at the time I thought I was helping (Former volunteer).

Our reflections became critical of the way some prison peer support schemes are operationalised, leading to anxiety that our findings could damage schemes – or result in reduced provision – and prisoners would suffer. Peer supporters face extremely high levels of distress and self-harm and, particularly given the chronic lack of other services such as healthcare (Testoni et al., 2021), their work is essential:

Those girls that I used to go out to, they used to slash their faces all the time, it really upset me, I used to say to them ‘*you’ve got your whole life ahead of you, don’t slash your beautiful face’* … the thought of removing this one thing (Listening), this one person that they have, to talk to, and that they feel safe with … I don’t know ….

Prisoners were motivated to work amidst difficult conditions to address unmet needs. They also described a push factor from prison:

*Former volunteer:* There's that power dynamic [of Incentives and Earned Privileges] in peer support roles, you are actually put under pressure …. If you read the landscape, you realise to get parole or … get out of this prison and see my kids, [I need] something good on my record …. So that must subconsciously influence the choice of roles … do providers know that? That there is this pressure to go into those roles?

We do not advocate peer support services being withdrawn. Yet, the risky contexts many peer supporters work in, often with insufficient training, support and services for the most unwell, pose a dilemma. We reflected on whether peer supporters should be doing this work at all, given that it risks offsetting prisons’ responsibilities for distressed prisoners onto prisoners themselves, compounding distress and deflecting attention from the need for structural reforms by applying a sticking plaster. At the same time, peer supporters offer vital places of safety within environments which can feel unsafe, uncaring, and devoid of trust:

Former volunteer: They just needed someone to talk to, and to show a bit of empathy …. It's really needed. No-one trusts officers and so you need that peer aspect, but then you have to support that.

Whilst the statement ‘no-one trusts officers’ does not apply to all prisoner-staff relationships, prisoners enforce a social code in defiance of official rules (Trammell, 2012) and co-authors reflected on difficult relationships with some prison staff. We therefore concluded that peer support serves an important function, offering isolated people, caught up in detention, someone to talk to and alleviating distress. However, peer support cannot be separated from broader questions including how many people (experiencing severe mental illness) we imprison (Tomczak and Bennett, 2020), how prisons are resourced and run and the adequacy of services relative to prisoner needs. It is concerning that peer workers are increasingly the *only* service available to prisoners experiencing mental ill-health, self-harm and/or suicidal intention. Voluntary organisations that coordinate peer support could harness their potential to regulate prisons (Tomczak, 2022b) by supplementing service delivery with acknowledgment of risky conditions and campaigning for structural reforms (Tomczak and Buck, 2019).

## Recommendations

We have offered a new perspective on prison peer supporters: as crucial, often life-saving actors working in extraordinarily difficult and often unreasonable conditions, who have *unique insight into the prison mental health crisis* and who require organisational support. Reflecting our focus on lived experience, this section draws on former prisoner strategies for service development.

Our first recommendation is that safe opportunities for suicidal and distressed prisoners to be listened to should not sacrifice peer supporters’ well-being. Policymakers and voluntary organisations must recognise that volunteering whilst imprisoned is significantly different from community volunteering and seek to learn from people with lived experiences to shape services. Imprisoned volunteers are often bullied and/or exploited. They cannot seek comfort from loved ones or go for a walk after a difficult ‘shift’ as their ‘home’ and ‘work’ spaces are fused, and they can struggle to step back from their role when burdened by prisoner distress (Jaffe, 2012). At minimum, prisoner volunteers require respect and understanding from staff, safe working conditions and supervisory support.

Our second recommendation is for policymakers and providers to acknowledge and work to reduce the potential for secondary and vicarious trauma within peer support. Comprehensive volunteer training could introduce these concepts so that prisoners can make informed decisions about taking part. This is especially important given the prevalence of trauma amongst prisoners (Facer-Irwin et al., 2022). We also advocate that volunteers and prison officers be trained in trauma-informed principles (e.g., Bradley, 2017; Levenson, 2020), including how to respond sensitively to volunteers witnessing self-inflicted harm and death. Supervision meetings (not attended by officers) could facilitate debriefing and improve therapeutic interactions (Powers, 2017), safeguarding volunteers and recipients. Ideally, supervisors should understand the complexities of prison: signposts to helplines are only useful if volunteers can access phones and spaces for confidential discussions. Finally, as the primary predictor of secondary trauma is hours-per-week spent working with traumatised people, (Bober and Regehr, 2006) all volunteers should have a line manager who ensures safe working hours.

Our third recommendation is for voluntary organisations to regularly review volunteer experiences and mobilise this knowledge to stimulate changes in and beyond prisons. We have shared findings with one voluntary organisation and will share findings with more stakeholders. However, there is an opportunity for *all* peer support providers to develop forums to learn from lived experience and strategically plan and act. (Former) prisoners can offer valuable local knowledge (Fals Borda, 1988) and viable solutions (Peralta, 2017), which may be overlooked by others. Collaborations with (former) prisoners could develop services fit for purpose in their context. Peer supporters and providers could seek to influence qualitative changes, such as all volunteers having safe, clean spaces to work in; confidential supervision; and places to wash and receive support following incidents.

More broadly, peer providers could couple service delivery with ‘upstream’ pressure for structural reforms (Quinn and Tomczak, 2021). Voluntary organisations are well placed to broker systemic change (Tomczak and Buck, 2019), for example, prison peer supporters could catalog callout types with their employing organisation (e.g., self-harm/severe mental health episode), who could feed these insights into multi-agency planning/commissioning forums locally and nationally. Regular meetings between providers and volunteers (e.g., quarterly safeguarding reviews) could add (anonymised) context and facilitate coproduced action plans. Peer support providers could then feed levels of need to those monitoring deaths in prison such as (in England) the Independent Advisory Panel on Deaths in Custody^4^ and the parliamentary Justice Committee, or (globally) Penal Reform International^5^, influencing policy on an (inter)national scale.

Critiques of coproduction argue that participants have little power to challenge or ‘change policy, neoliberal economic structures or austerity drives’, however, involving national social movements and policymakers ‘may support a wider force for change, illuminating different possible paths for the development of public services’ (Farr, 2018: 640). Anonymised records of (unmet) needs could align with campaigns to raise awareness of the scale of the prison mental health crisis and the unsuitability of prison as a ‘place of safety’ (Tomczak, 2022a), enabling voluntary sector actors to advocate for community alternatives to imprisonment and more comprehensive mental health provision within *and outside* prisons.

### Limitations

Our sample size was small, based on the experiences of five volunteers in England during the last decade, to provide richly textured data and ensure co-authors were supported. However, this means our findings are not generalisable. Most team members wanted this article to include their real names but decided not to attribute specific details or accounts to themselves to offer some anonymity. This means we have not included details about individual prisons, sentences, suicide prevention initiatives, or time spent as peer supporters. Whilst the emotional harm we document remains valid regardless of setting or level of experience, future research could use larger samples and examine different prisons, charitable organisations and settings outside of England.

## Conclusion

Deteriorating prison safety poses a major moral, social, economic and public health threat. The consequences of unsafe prisons are absorbed by societies. Of the 78,000 prisoners in England and Wales, fewer than 100 will never be released (Prison Reform Trust, 2021). Rising prison populations, staff reductions and significant increases in suicide and self-harm indicate that prisons are now less safe than at any point in recorded history. Using peer supporters as a major part of the response to this crisis (Gauke, 2018), problematically relies on imprisoned volunteers working in dangerous and distressing situations with minimal support.

The data from this exploratory study reveal the human impacts of such policies. Whilst most studies of prison peer support engage serving prisoners, highlighting the potential to support desistance and improve relationships with prison staff (Einat, 2017; Perrin et al., 2018), we highlight that peer supporters can experience major problems. Our innovative, participatory, retrospective design created space for former volunteers to reflect on the unique risks of peer support that can sometimes be overlooked or subsumed in more appreciative accounts, including fraught relationships, high expectations, limited support, and destabilising pressures. The analysis illustrated inconsistencies in training and working conditions, which can leave people ill-equipped for the levels of distress they encounter. Prisoners were often called to people experiencing acute mental health crises, who require professional healthcare. This brought awareness of the risks of secondary and vicarious trauma and generated optimism that organisations could better meet volunteer needs whilst steering health and justice policymakers toward more comprehensive mental health provision and diversion into healthcare. We reflected on the need for voluntary providers to highlight gaps in provision and avoid concealing problematic carceral practices and structural inequalities through sticking plaster solutions (Carlton and Scraton 2017).

We ended by discussing the value of peer support in prisons, given its unique ability to offer isolated and traumatised people someone to talk to and alleviate distress. Our solutions cultivated commitment to the therapeutic and safeguarding features of peer support alongside the belief that much more comprehensive mental health provision is required in (and in many cases instead of) prisons. Peer supporters are ideally placed to illustrate the unacceptable scale of the prison mental health crisis, enabling providers to advocate for alternatives to imprisonment, more suitable and comprehensive mental health services, and reductions in the structural inequalities that produce criminalisation and ill-health.

## Notes

1.
https://reliefweb.int/report/world/un-expert-states-have-duty-probe-all-suspicious-deaths

2. The ‘Listener suite’ is normally a double cell, where a prisoner can talk confidentially to a Listener, and if necessary, spend the night, yet availability and quality are inconsistent. HMIP (2016: 12) found suites were not available at every prison, some lacked furnishings, some were dirty or damaged and one suite was ‘blood-spattered’.

3. Segregation units, known colloquially as the seg, are generally located deep inside the main body of a jail with 23 out of 24 h spent in solitary confinement (James, 2015).

4. This non-departmental public body provides independent advice to the U.K. Ministerial Board on Deaths in Custody aiming to prevent deaths in custody. https://www.iapondeathsincustody.org/

5.
https://www.penalreform.org/resource/deaths-in-prison-examining-causes-responses-and-prevention/
